# Thixotropic Supramolecular Gel Based on l-Lysine Derivatives

**DOI:** 10.3390/gels1010081

**Published:** 2015-07-31

**Authors:** Masahiro Suzuki, Yuta Hayakawa, Kenji Hanabusa

**Affiliations:** Graduate School of Science and Technology, Shinshu University, 3-15-1 Tokida, Ueda, Nagano 386-8567, Japan; E-Mails: 13fm626c@shinshu-u.ac.jp (Y.H.); hanaken@shinshu-u.ac.jp (K.H.)

**Keywords:** organogels, l-lysine, gelation, thixotropy, nanofibers, supramolecular chemistry

## Abstract

The dimer l-lysine derivatives, in which two *N*^α^,*N*^ε^-diacyl-l-lysines were crosslinked by calcium ion, were synthesized through a simply synthetic procedure and their gelation properties were examined. These compounds functioned as an organogelator; especially, the gelators possessing both a linear and a branched alkyl chains had the better organogelation ability and formed the thermally stable and rigid organogel. In addition, some organogels had a thixotropic property, which were responsive to a mechanical stimulus and reversibly underwent the gel–sol transition at room temperature. The thixotropic behavior was confirmed by visual contact and rheological experiments. Furthermore, it was assumed the mechanism of the thixotropic behavior.

## 1. Introduction

There has been a great study on a low-molecular-weight gelator and its gel (supramolecular gel) over the past decades, because of its academic interests and potential applications to cosmetics, foods, medical and pharmaceutical, photonic and electronic devices [1,2,3,4,5,6,7,8,9,10]. Although many low-molecular-weight gelators have been discovered and a few low-molecular-weight gelators have been used in cosmetics and commodities, their market shares are small, compared with polymer gelators.

On the other hand, one of the recent research trends in low-molecular-weight gelators is the development of supramolecular gels with one or more functionality. Especially, a control of the isothermal gel-to-sol and sol-to-gel transition by external stimuli, such as sonication [11], photo-irradiation [12,13], pH [14], enzyme [15], ions [16], shear force [17], and redox [18], has been investigated. A thixotropic supramolecular gel, which repeatedly undergoes the gel-to-sol transition by shearing and then sol-to-gel transition by standing, is a promising material. In spite of the many needs from industrial fields, it is very difficult to prepare such a gel. A common supramolecular gelation is similar to the mechanism of crystallization, after dissolution of gelators by heating, then the promotion of self-assembly by cooling. The energy gap between heating and cooling is very important for the promotion of self-assembly. In addition, the gelator molecules randomly self-assemble into the three-dimensional networks, involving the formation of nanofibers (one-dimensional aggregates). Therefore, the collapsed gel never regenerates at room temperature [19]. Although some thixotropic supramolecular gels have been reported [20,21,22,23,24,25], thixotropic supramolecular gels are serendipitously discovered.

In order to prepare the thixotropic supramolecular gels, some strategies have been reported. Weiss *et al.* reported the thixotropic organogels formed by the spherulitic self-assembled fibrillar networks [21]. Shinkai *et al.* prepared the thixotropic gels that the gelators self-assembled into disk-like aggregates [26]. The alkylurea organogels with “house of cards” structure composed of tape-like and sheet-like aggregates showed the thixotropic property [27]. Pochan *et al.* reported the thixotropic hydrogel based on β–hairpin peptides and their fast recovery times from sol to gel [28]. We previously reported the thixotropic supramolecular gels using cyclodipeptide derivatives as the gelators [29,30]. The gelators form a one-dimensional aggregate (supramolecular polymer) through strong hydrogen bonding interaction between the cyclodipeptide segments and create a three-dimensional network by weak interaction of van der Waals force between the side chain segments; the shearing breaks the weak van der Waals interaction (but not the strong hydrogen bonding interaction), and the gel readily collapses. When the sol sample is allowed to stand, three-dimensional networks regenerate through the weak van der Waals interaction, leading to the re-formation of the gel. In this paper, we describe the supramolecular organogelation of new l-lysine-based low-molecular-weight gelators and thixotropic properties of the supramolecular gels.

## 2. Results and Discussion

### 2.1. Synthesis

**Figure 1:**
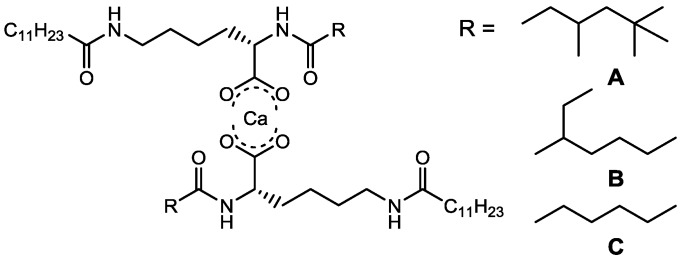
Chemical structures of l-lysine derivatives.

These compounds were prepared by the simply synthetic procedure. When an aqueous solution of CaCl_2_ (0.1 M) was added to an aqueous solution of *N*^α^-alkyl-*N*^ε^-dodecyl-l-lysine sodium salt with stirring, the product was immediately obtained as a white precipitate. As reported previously, the l-lysine derivatives (sodium salt) were also obtained; the commercial *N*^ε^-dodecyl-l-lysine and acid chlorides were mixed, with the high yield [31]. The gelators, which can be synthesized in the simple procedure with high yield, should be suitable for practical uses. The chemical structures are shown in Figure 1.

### 2.2. Gelation Test

The gelation test was performed by a test tube inversion method. The results of the gelation tests of gelators A–C are listed in Table 1 [32]. Gelators A and B functioned as a good organogelator in organic fluids and had a similar organogelation ability, while gelator C had a low organogelation ability. Gelator C trended to be low solubility and showed “precipitate” or “insoluble” for many organic fluids, although it formed toluene gel and chloroform gel at 1 wt%. In contrast, gelators A and B dissolved in organic fluids at lower heating temperature and had the similar gelation ability; gelators A and B were easier to handle for the gel formation. These results indicate that the alkyl group at *N*^α^ position on the lysine plays an important role in the organogelation (and solubility).

**Table 1:** Results of gelation tests.

Solvent	A	B	C
n-Hexane	GT(6) *	GT(8) *	I
n-Dodecane	GT(8) *	GTL(20)	P
Liquid paraffin	S	P	P
Squalane	P	P	P
Cyclohexane	GT(20) *	GT(6) *	I
Ethanol	GO(30)	P	GO(20)
Ethylene glycol	GO(30)	GO(30) *	–
IPM	GT(20)	GTL(10)	–
Toluene	GT(80) *	GT(15) *	GT(10)
Chloroform	GT(20) *	GTL(30) *	GT(10)
Castor oil	GT(40)	GT(80)	GT(80)
Kerosene	GT(15) *	GTL(10) *	GTL(40)
Light oil	GT(20) *	GT(6) *	GTL(40)

Values denote minimum gel concentration (MGC, mg/mL). GT: transparent gel; GTL: translucent gel; GO: opaque gel; I: almost insoluble; P: precipitate; IPM: isopropyl myristate; *: thixotropic gel.

### 2.3. Morphology, Gel Strength and Gel-to-Sol Transition Temperature (T_gel_)

**Figure 2:**
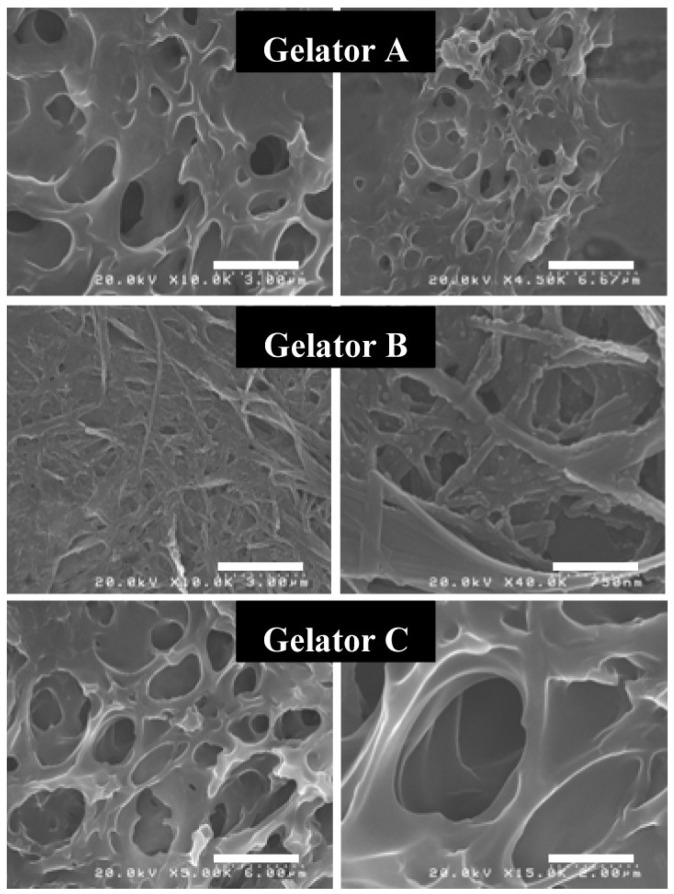
FE-SEM images of dried samples prepared from kerosene gels based on gelator A, gelator B and gelator C. Scale bars: Gelator A (left: 3 μm, right: 6.67 μm); Gelator B (left: 3 μm, right: 750 nm); Gelator C (left: 6 μm, right: 2 μm).

The nanostructure of the gelators formed in the supramolecular gels was evaluated by a field-emission scanning electron microscope (FE-SEM). Figure 2 shows the FE-SEM images of the dried samples prepared from the kerosene gels. These gelators created a three-dimensional network by entangling of the self-assembled nanofibers. The organogels are formed by trapping organic solvents into spaces in the three-dimensional networks. Gelators A and C formed the similar nanostructure, while gelator B created the closer networks. For other solvents, for example, such as cyclohexane and chloroform, the three-dimensional networks created by the self-assembled nanofibers were observed [32]. In addition, the Fourier Transform Infrared (FT-IR) study detected the typical peaks that were arisen from the hydrogen bonded amide groups and the alkyl groups with van der Waals interaction [32,33]; therefore, the driving forces for the organogelation were hydrogen bonding and van der Waals interactions.

**Figure 3:**
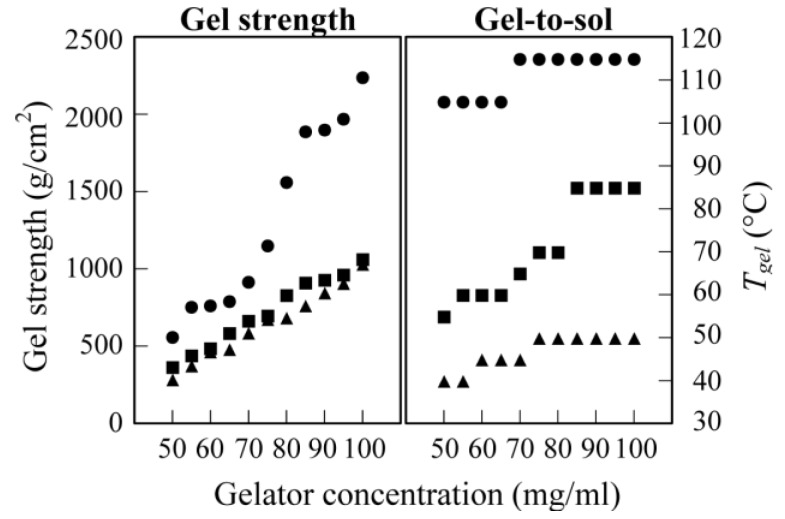
Gel strength (**left**) and thermal stability (*T*_gel_ (**right**)) of kerosene gels based on gelator A (■), gelator B (●) and gelator C (▲).

The properties of the supramolecular gels were evaluated by their mechanical and thermal stabilities. Figure 3 shows the dependence of the gel strength and the gel-to-sol transition temperature (*T*_gel_) of the kerosene gels on the gelator concentration. The gel strength increased with the increasing concentration of the gelator. This is attributed to the fact that the gel networks become closer with the increasing concentration of the gelators. Gelators A and C has the similar gel strength, while gelator B formed the more rigid gel; e.g., at 100 mg/mL, the gel strengths were 1065 g/cm^2^ for gelator A, 900 g/cm^2^ for gelator C, and 2240 g/cm^2^ for gelator B (the gel strength is *ca*. twice as rigid as other gels). As mentioned above, gelator B created the close networks compared with gelators A and C forming the similar network structure. Such the close network structures may bring about the large gel strength.

Kerosene gel of gelator B had the excellent thermal stability and retained its gel state at 100 °C. In contrast, gelator C could not form the gel at more than 50 °C. These facts indicate that the gelators with a branched alkyl chain form a thermally stable gel.

### 2.4. Simple Thixotropy Test of Supramolecular Gels

**Scheme 1:**
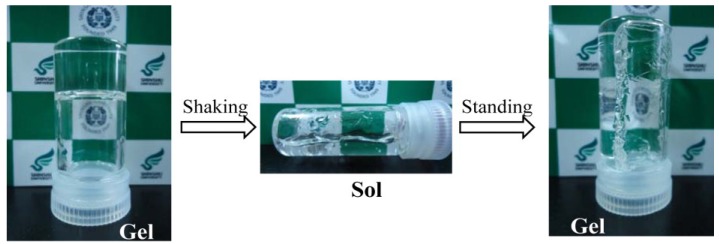
Thixotropic behavior of kerosene gel based on gelator A.

For these supramolecular gels, the simple thixotropy test was carried out as follows: the gel formed in the gelation test was broken by a shaker or a spatula and then the sample was allowed to stand at 25 °C for 2 h. If the gel was re-formed, even when repeatedly undergoing the five operations, the gel was considered to be a “thixotropic gel”. In Table 1, the gels with “*” are a thixotropic gel. Gelator C did not form a thixotropic gel. On the other hand, most of the gels formed by gelators A and B had a thixotropic property (although they did not form the thixotropic gel for ester oil (IPM) and castor oil). Typical thixotropic behavior is shown in Scheme 1. When the kerosene gel was shaken, the gel-to-sol transition readily occurred. After standing the sample at 25 °C for 2 h, the gel completely regenerated.

### 2.5. Rheological Studies

Thixotropic properties of these supramolecular gels, which underwent the reversible phase transitions (gel-to-sol by shaking and sol-to-gel by standing) at room temperature, were evaluated further by using some rheological experiments. In general, the evidence for the thixotropic property can be obtained by a step-strain time dependent rheological analysis and shear stress–shear rate dependent rheological analysis (flow curve): the former is an observation of a recovery of gel after shearing, the latter is an appearance of a hysteresis loop.

As the preliminary experiments, we conducted a frequency sweep and strain sweep rheological analyses of kerosene gels. In the plots of storage modulus (G′) and loss modulus (G′′) against frequency or strain [32], G′ was larger than G′′ at the gel state. When the frequency and strain increased and the gel changed into the sol state (collapsed), G′′ becomes larger than G′. From these experimental results, the maximum values of strain and frequency, which were able to retain the gel states, obtained; the maximum frequencies for all kerosene gels were less than or equal to 4 Hz, and the maximum strains were 0.4% for gelator A, 2% for gelator B and 0.1% for gelator C. The fact that the kerosene gel of gelator B has the largest value of the maximum strain compared with other gelators is agreed with the result of the gel strength.

**Figure 4:**
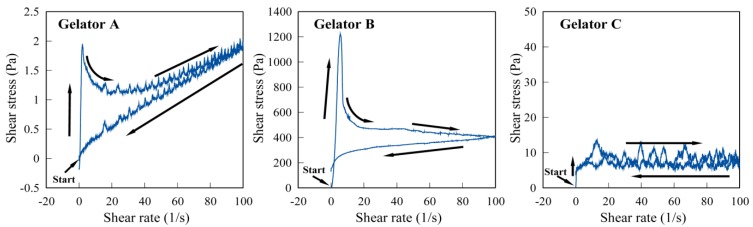
Flow curves of kerosene gels based on gelator A (10 mg/mL), gelator B (15 mg/mL) and gelator C (50 mg/mL).

To evaluate the thixotropic property of the supramolecular gels, their flow curves were first measured. The measurement was carried out as follows: after collapsing of the gel by shearing, and then the sample was allowed to stand for 30 min. The shear stress was measured when increasing the shear rate from 0 to 100 s^–1^ and then decreased to 0 s^–1^. Figure 4 shows the typical flow curves of kerosene gels based on gelators A–C. For the kerosene gels of gelators A and B, the shear stress increased with increasing shear rate (the gel collapsed) and then decreased. The shear stress decreased during the decreasing shear rate. It is noted that the hysteresis loop making a clockwise turn was observed; *i.e.*, the changing of the shear stress with decreasing the shear rate passed through the different route from that with increasing the shear rate. Such the observation of the hysteresis loop is one of the obvious evidence that the gel has a thixotropic property. The similar hysteresis loops were observed for all thixotropic gels in listed in Table 1 (data not shown). In contrast, no hysteresis loops were observed for the gels based on gelator C, indicating that the gel hardly had the thixotropic property (agreed with the result of the simple thixotropy test).

**Figure 5:**
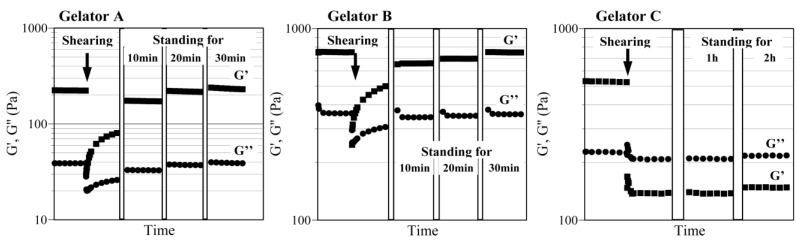
Time dependent rheological analysis of kerosene gels based on gelator A (10 mg/mL), gelator B (15 mg/mL) and gelator C (50 mg/mL) at a fixed strain (0.05%) and frequency (0.05 Hz).

For a thixotropic gel, its recovery time of the sol-to-gel transition after shearing is one of the important properties. The recovery time was observed by the time dependent rheological analysis. The shearing to collapse the gel was carried out at 40% strain. Figure 5 shows the time dependent rheological analysis of kerosene gels based on gelators A–C at a fixed strain (0.05%) and frequency (0.05 Hz). For gelator C (no formation of thixotropic gel), the values of G′ and G′′ sharply decreased just after shearing and it was changed from G′ > G′′ into G′ < G′′ (the gel collapsed). The sample maintained the G′ < G′′ even when standing for 120 min. In contrast, the kerosene gels of gelators A and B collapsed just after shearing (G′ < G′′) and it was changed into G′ > G′′ (the gel re-formed) within several seconds. The G′ and G′′ values completely recovered by standing for 30 min. These results obviously indicate that the kerosene gels based on gelators A and B have a thixotropic property. Almost the same rheological results were obtained for the same operation repeatedly conducted over 300 times [32].

### 2.6. Appearance of Thixotropic Property

**Figure 6:**
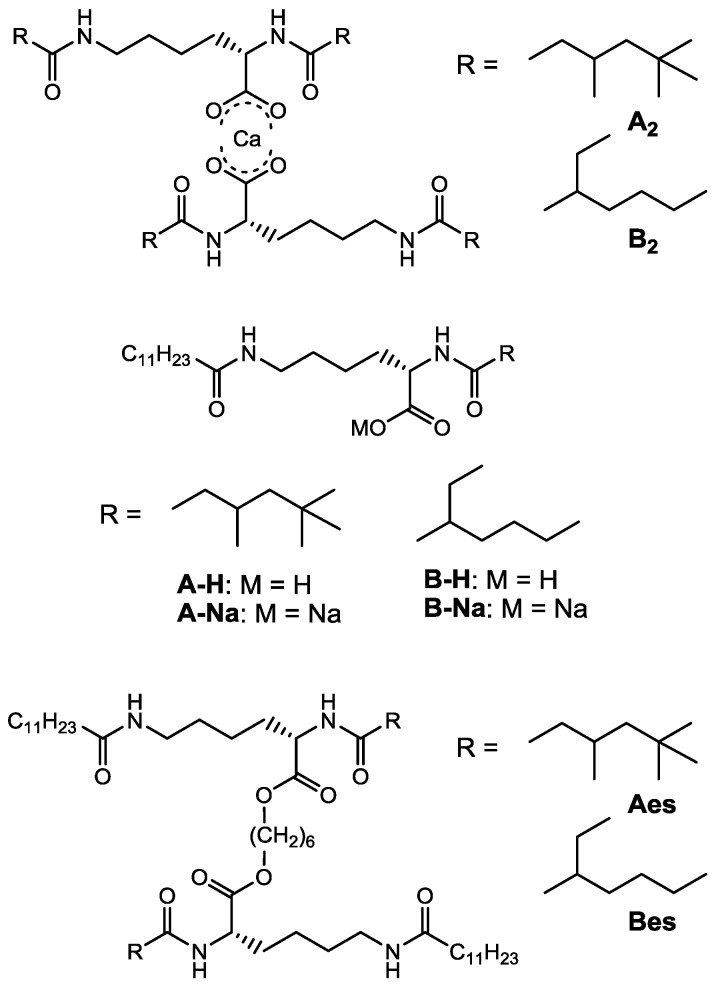
Various l-lysine-based gelators.

To consider the appearance of the thixotropic property, some l-lysine analogues of gelators A and B as shown in Figure 6 were synthesized and their thixotropic properties were examined. Compounds A_2_ and B_2_ have a dimer structure, in which two diacyl-l-lysines with the same branched alkyl chains are crosslinked by calcium ion. Gelators A-Na and B-Na are a monomer gelator with a charge (ionic monomer gelator). Gelators A-H and B-H are a non-charged monomer gelator. However, gelator A-H forms a dimer structure in the gel through the hydrogen bonding interaction between carboxylic acid groups (semi-ionic dimer gelator), while a portion of gelator B-H forms a dimer structure [34]. Gelators Aes and Bes are a non-ionic dimer gelator, in which two diacyl-l-lysines are crosslinked by the alkylene spacer through an ester bond (covalent bond).

In the present system, it can be presumed that the thixotropic property is induced by some factors; e.g., charges, dimer structures, branched alkyl groups, *etc.* Although compounds A_2_ and B_2_ were more simply synthesized than gelators A and B, they were well-soluble in many organic fluids and hardly functioned as an organogelator. In addition, the fact that gelator C did not form the thixotropic gel indicates the necessity of both the linear and branched alkyl chains. The simple thixotropy test of kerosene gels based on these gelators demonstrated that gelators A-H, A-Na, and B-Na formed the thixotropic gels, while the gels of gelators B-H, Aes and Bes did not re-formed after collapsing by the shaking even when standing 24 h. The rheological evidence for the thixotropy was supported by the hysteresis loops [32]. Furthermore, the time dependent rheological analyses demonstrated the recovery time of their gels was slow compared with the gels based on gelators A and B; 5 h for gelator A-H, 2 h for gelator A-Na, and 4 h for gelator B-Na.

These results give us some important information on the appearance of thixotropic property, the effects of dimerization, and charge. Compared with gelator A-H (semi-ionic dimer structure), gelator A-Na (ionic monomer gelator) forms the thixotropic gel with fast recovery time, and gelator B-H (semi-ionic monomer) and gelators Aes and Bes (non-ionic dimer gelator) does not re-form the gel. The appearance of thixotropic property in gelators A and B is induced by the cooperative effects of the charge (calcium ion) and the dimer structure in addition to the introduction of both the linear and branched alkyl chains.

### 2.7. Mechanism of Thixotropic Behavior

Though there is the rheological evidence that these gels have the thixotropic property, it is very difficult to analyze the mechanism of the thixotropic behavior. For example, the electron microscopic analyses [Transmission electron microscopy (TEM) and SEM] for the dried gels prepared from the kerosene gels before and after shearing demonstrated the similar nanostructures (as shown in Figure 2), because the nanofibers may rearrange during the drying process. The powder X-ray diffraction is almost the same situation as the electron microscopy. The Nuclear Magnetic Resonance (NMR) spectroscopy was also not observed the difference. In addition, other spectroscopic analyses, such as UV-Vis, circular dichroism (CD) and fluorescence, did not show any different situations [32].

Fortunately, the slightly different results were obtained in the FTIR spectroscopy and Atomic Force microscope (AFM) study. The nanofibers in the gels form through a hydrogen bonding interaction between the amide groups and van der Waals interaction between the alkyl chains [33]. The FTIR peaks of the hydrogen bonded amide groups hardly changed by shearing, while the IR region (2850–2930 cm^–1^) originating from the alkyl chains (methylene) slightly changed [32]. The experimental results indicate that the gel-to-sol transition is induced by the change in the alkyl chains (but not change in the hydrogen bonding interaction). In addition, the complex IR spectra and the slight change may indicate that the two kinds of van der Waals interactions, strong and weak interactions. The strong interaction is mainly the interaction between the linear alkyl chains and participates in the formation of self-assembled nanofibers involving hydrogen bonding. In contrast, the weak interaction is mainly the interaction between the blanched alkyl chains and participates in the creation of the three dimensional networks. Though the interaction between the branched and linear alkyl chains is also considered, it may be involved in the weak interaction due to the large van der Waals radius of the branched alkyl chains. Probably, the slight IR change and the low density of three-dimensional networks by shearing are induced by the destruction of the weak van der Waals interaction (Scheme S1) [32].

**Scheme 2:**
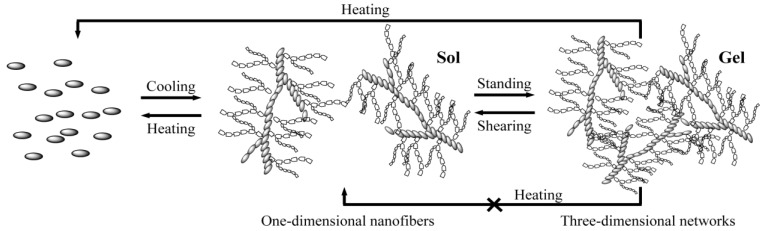
Tentative illustration of gelation and thixotropic behavior at room temperature.

On the basis of these results, we propose the mechanism of organogelation and reversible phase transition at room temperature is similar to the colloidal gel shear-thinning mechanism [28] and the tentative illustration is shown in Scheme 2. For the gelation, the gelator molecules form the one-dimensional nanofibers self-assembled through hydrogen bonding between the amide groups and strong van der Waals interaction between the linear alkyl chains, and the one-dimensional nanofibers create the three-dimensional networks by physical crosslinking through the weak van der Waals interaction between the branched alkyl chains. By shearing, physical crosslinks (weak van der Waals interactions) are disrupted (*i.e.*, some entanglements of nanofibers are destroyed), but not the one-dimensional nanofibers because the strong van der Waals and hydrogen bonding interactions are maintained. As the result, the nanofibers separate from the networks, and the gel changes into the sol. The physical crosslinks are constructed again during the standing, and the three dimensional networks are re-created. The physical crosslinking and destroying occurs reversibly at room temperature. It is noteworthy that such a change, from three-dimensional networks into one-dimensional nanofiber, cannot be controlled by temperature.

## 3. Conclusions

In conclusion, we revealed that new l-lysine-based gelators, which had a dimer structure consisting of two *N*^α^,*N*^ε^-diacyl-l-lysines crosslinked by calcium ion, formed the thixotropic organogels. These gelators were simply synthesized and functioned as a good organogelator. Especially, the organogels based on gelators with both the linear and branched alkyl chains had a thixotropic property, which was confirmed by the rheological evidence. The results of some l-lysine analogues indicate that at least three factors, the charge, the dimer structure, and possession of both the linear and branched alkyl chains, are contributed to the appearance of thixotropic property. Furthermore, it is assumed that the reversible gel-to-sol transition at room temperature (thixotropy) is induced by the on–off of the weak van der Waals interaction between the branched alkyl chains. A further study of the thixotropic supramolecular gels, such as controlling of recovery time, molecular designs of more simply structured gelators, and the detailed mechanism are now in progress.

## 4. Experimental Section

### 4.1. Materials

*N*^ε^-Dodecyl-l-lysine was obtained from the Ajinomoto. The other chemicals were of the highest grade commercially available and used without further purification. All solvents used in the syntheses were purified, dried, or freshly distilled as required. The *N*^α^-alkyl-*N*^ε^-dodecyl-l-lysines were synthesized according to the literature [30].

### 4.2. Apparatus for Measurements

The elemental analyses were performed on a Perkin-Elmer series II CHNS/O analyzer 2400. The FTIR spectra were recorded on a JASCO FS-420 spectrometer. The field emission scanning electron microscope (FE-SEM) observations were carried out using a Hitachi S-5000 field emission scanning electron microscope. The TEM images were obtained with a JEOL TEM-2010 electron microscope. The ^1^H-NMR spectra were measured by using a Bruker AVANCE 400 spectrometer with TMS as a standard. The gel strengths were measured by using a Sun Science Sun RheoMeter CR-500DX. The wide-angle X-ray diffraction (WAXD) measurements were performed by using a Rigaku 8Rad-rX diffractometer.

### 4.3. Gelation Test

The gelation properties for 40 different solvents and pure water were tested. The typical procedure for gelation testing is as follows: a weighed gelator is mixed with a solvent (1 mL) in a test tube with a screw cap (14-mm inner diameter) and heated until the solid is dissolved. The resulting organic solvent is cooled to room temperature (25 °C) for 2 h. Gelation is examined visually; when no fluid runs down the walls of the tube upon inversion, the material is considered to be a “gel”.

### 4.4. Gel Strength

Samples were prepared as follows: a mixture of a weighed gelator in solvent (2 mL) in a sealed sample tube (15 mm in diameter) was heated until a clear solution appeared. The resulting solution was allowed to stand at 25 °C for 6 h. The gel strength was evaluated as the force necessary to sink a cylinder bar (10 mm in diameter) 4 mm deep in the gel.

### 4.5. Gel-to-Sol Transition Temperature (T_gel_)

The experiment for gel-to-sol transition temperature (*T_gel_*) was carried out in an incubator with temperature control (±0.5 °C) using a test tube inversion method. A mixture of a weighed gelator in water (1 mL) in a sealed test tube was heated until a clear solution appeared. After allowing the solutions to stand at 25 °C for 4 h, the gel samples were allowed to stand in a temperature-controlled incubator. The incubator was repeatedly operated such that the temperature was elevated at 2 °C and then kept it for 30 min.

### 4.6. Rheological Analysis

The rheological measurements were performed on a NRM-2500 rheometer (Elquest Co. Ltd., Osaka, Japan) using a corn plate with 6.2 cm in diameter and θ = 3 degree in corn angle. A water bath was equipped for temperature control. The gel samples were prepared in the sample tube and then stood at 25 °C for 1 h. The sample was transferred onto the corn plate and measured at 25 °C.

### 4.7. FE-SEM

The dried gels were prepared as follows: the kerosene gels were frozen in liquid nitrogen, and then dried in vacuum for 48 h. The dried gels were shadowed to *ca.* 5 nm thick with Pt–Pd by sputtering. The dried samples were prepared by freeze-drying for cyclohexane gels and the room temperature drying for chloroform gels in vacuum.

## Supplementary Material

Supplementary information could be found: http://www.mdpi.com/2310-2861/1/01/0081/s1.
