# Supplementary material for: Thixotropic Supramolecular Gel Based on l-Lysine Derivatives
Source: Gels. 2015 Jul 31;1(1):81–93. doi: 10.3390/gels1010081 (PMC6318632; doi:10.3390/gels1010081)
Supplement: Supplementary file 1 [file gels-01-00081-s001.pdf]

Supplementary Information

1. Results of Gelation test. (Table S1).
2. Rheological results (Figures S1–S6).
3. Rheological data (Table S2).
4. FTIR spectrum (Figure S7).
5. AFM image (Figure S8).
6. CD spectra (Figure S9).
7. FE-SEM images (Figure S10).
8. Tentative interactions (Scheme S1).

Table S1. Results of gelation tests of A–D at 25°C.

Solvent	A	B	C
n-Hexane	GT(6) *	GT(8) *	I
n-Dodecane	GT(8) *	GTL(20)	P
Liquid paraffin	S	P	P
Squalane	P	P	P
Cyclohexane	GT(20) *	GT(6) *	I
Methanol	P	I	P
Ethanol	GO(30)	P	GO(20)
1-Propanol	P	S	S
Ethylene glycol	GO(30)	GO(30) *	–
Acetone	S	I	P
Cyclohexanone	S	P	S
Ethyl acetate	P	P	P
IPM	GT(20)	GTL10)	-
THF	S	GT(40) *	S
1,4-Dioxane	S	I	P
DMF	-	P	-
DMSO	S	S	P
Sulfolane	P	P	P
PC	P	P	P
γ–BL	P	P	P
Toluene	GT(80) *	GT(15) *	GT(10)
Chlorobenzene	-	GT(10) *	S
Nitrobenzene	P	P	P
Acetonitrile	I	I	I
Chloroform	GT(20) *	GTL(30) *	GT(10)
Linseed oil	VS	S	S
Castor oil	GT(40)	GT(80)	GT(80)
Si-oil (KF-54)	S	S	P
Si-oil (KF-56)	-	P	P
D4	I	I	I
D5	I	I	I
Kerosene	GT(15) *	GTL(10) *	GTL(40)
Light oil	GT(20) *	GT(6) *	GTL(40)
TEG	P	P	P
PPG700	GO(80)	GO(80)	P

Values denote minimum gel concentration (MGC, mg/mL). GT: transparent gel; GTL: translucent gel; GO: opaque gel; I: almost insoluble; P: precipitate; VS: viscous fluid. IPM: isopropyl myristate; THF: tetrahydrofuran; DMF: *N*,*N*-dimethylformamide; DMSO: dimethylsulfoxide; PC: propylene carbonate; γ–BL: γ–butyrolactone; Si-oil: silicone oil (Shin-etsu Chem.); D4: octamethylcyclotetrasiloxane; D5: decamethylcyclopentasiloxane; TEG: tetraethylene glycol; PPG700: poly(propylene glycol), MW = 700. *: thixotropic gel.

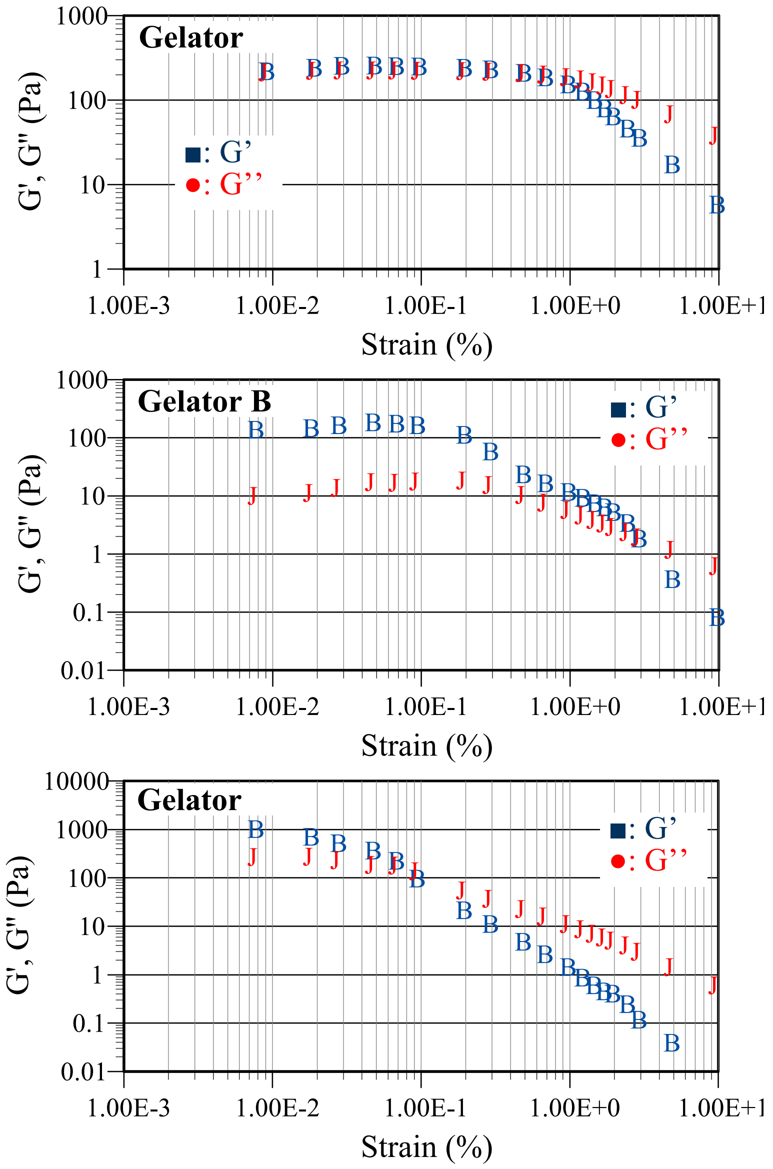

Figure S1. Strain sweep rheological analysis of the kerosene gels at a constant frequency (0.05 Hz). Gelator A = 10 mg/mL; Gelator B = 15 mg/mL, and Gelator C = 50 mg/mL.

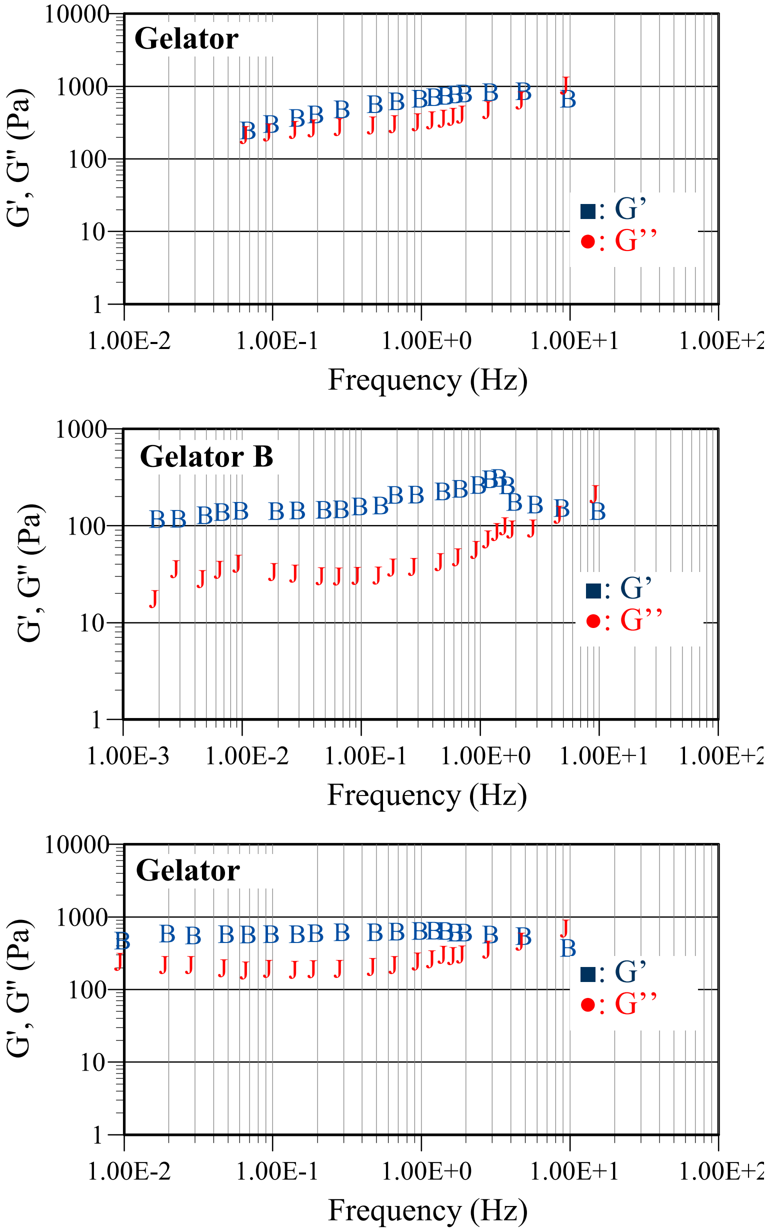

Figure S2. Frequency sweep rheological analysis of the kerosene gels at a constant strain (0.05 %).

Table S2. Rheological data for kerosene gels based on gelators A–C.

	**Gel (G′ > G″)**		**Sol (G′ < G″)**	
	Strain (%)	Frequency (Hz)	Strain (%)	Frequency (Hz)
Gelator A	≤ 0.4	≤ 4	> 0.4	> 4
Gelator B	≤ 2.0	≤ 4	> 2.0	> 4
Gelator C	≤ 0.1	≤ 4	> 0.1	> 4

Kerosene gels: [gelator A] = 10 mg/mL; [gelator B] = 15 mg/mL; [gelator C] = 50 mg/mL.

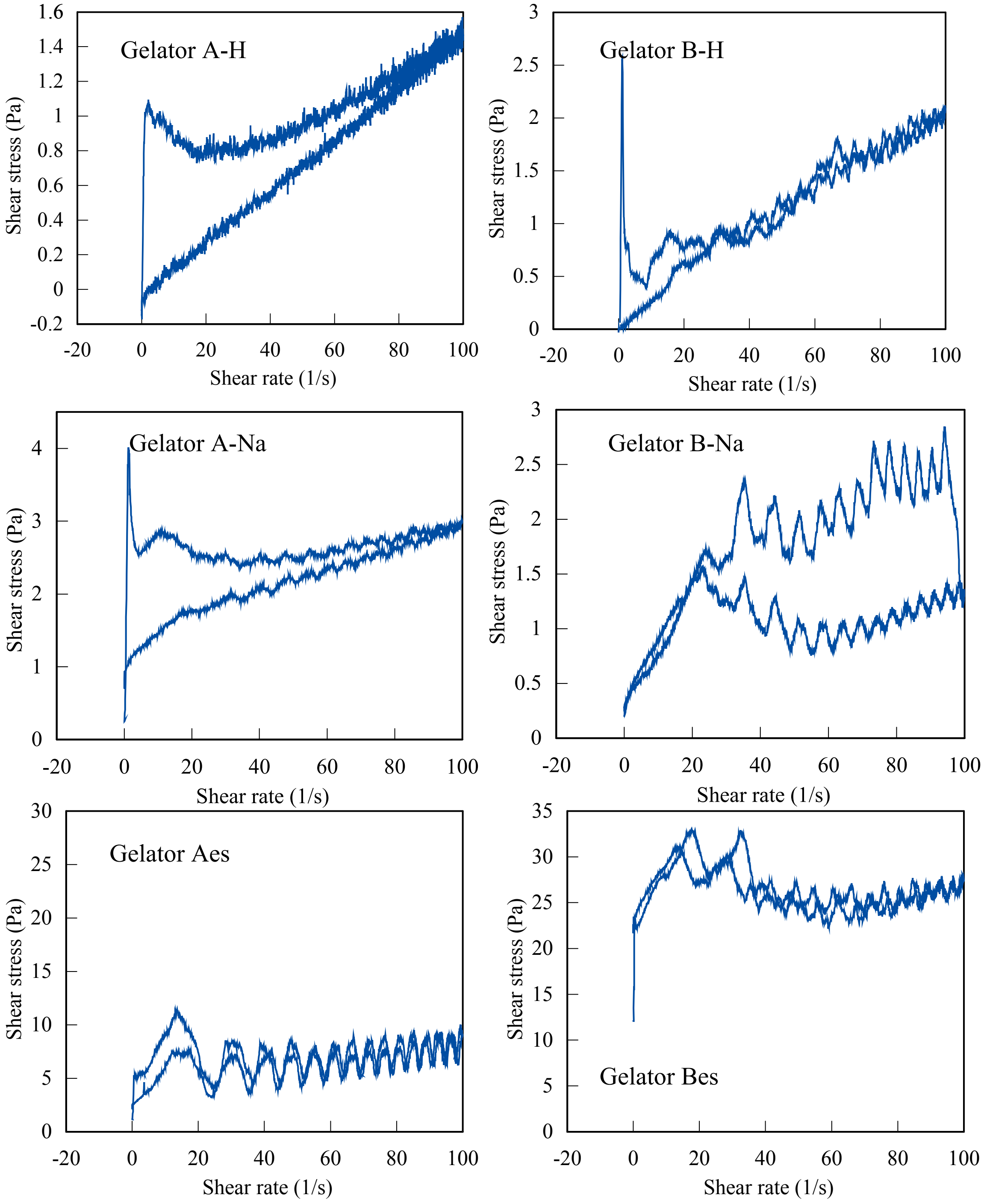

Figure S3. Flow curves of kerosene gels based on gelator A-H (10 mg/mL), gelator A-Na (8 mg/mL), gelator Aes (8 mg/mL), gelator B-H (6 mg/mL), gelator B-Na (8 mg/mL), and gelator Bes (10 mg/mL).

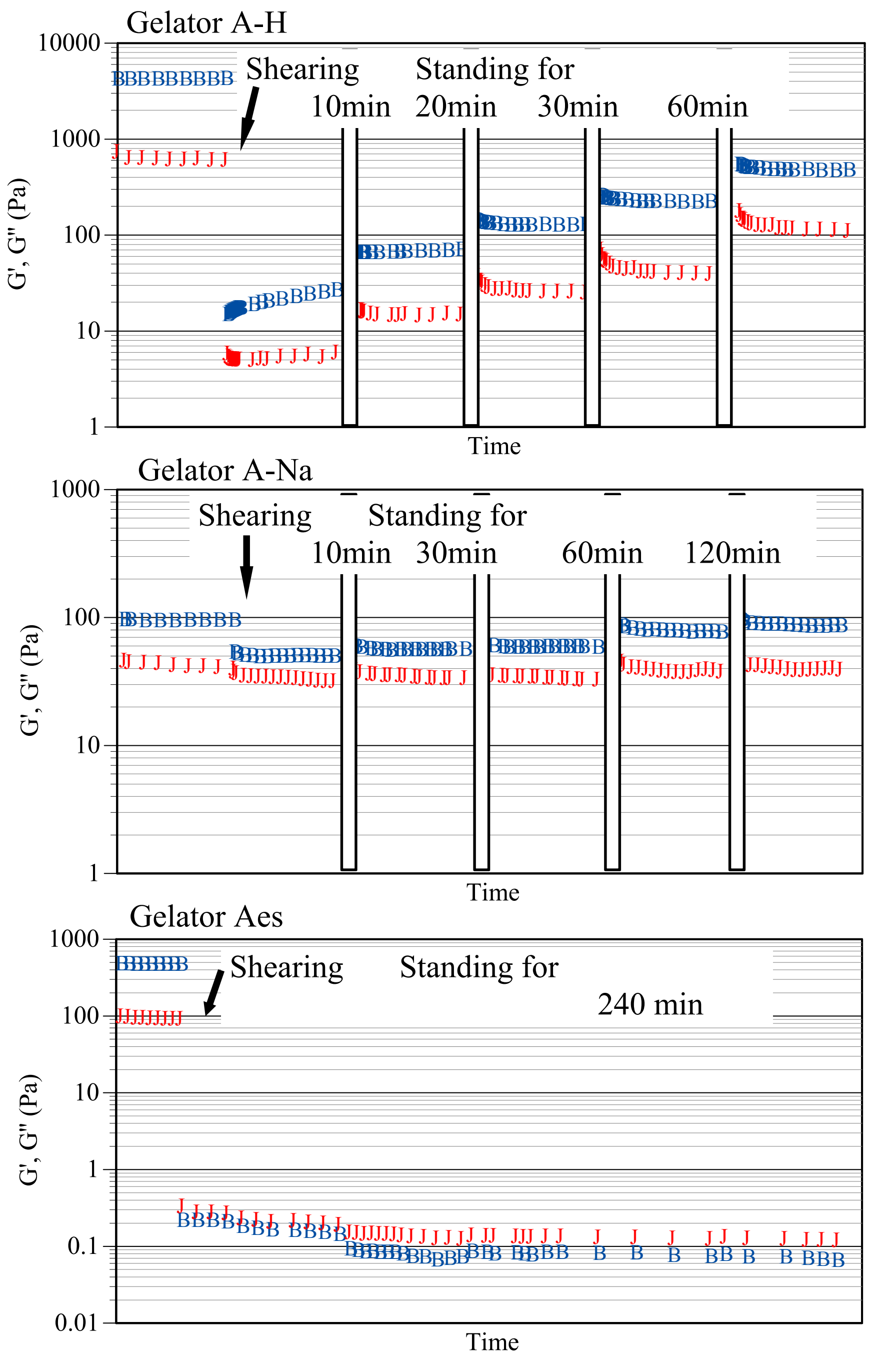

Figure S4. Time dependent rheological analysis of kerosene gels based on gelator A-H (10 mg/mL), gelator A-Na (8 mg/mL), gelator Aes (8 mg/mL), at a fixed strain (0.05%) and frequency (0.05 Hz).

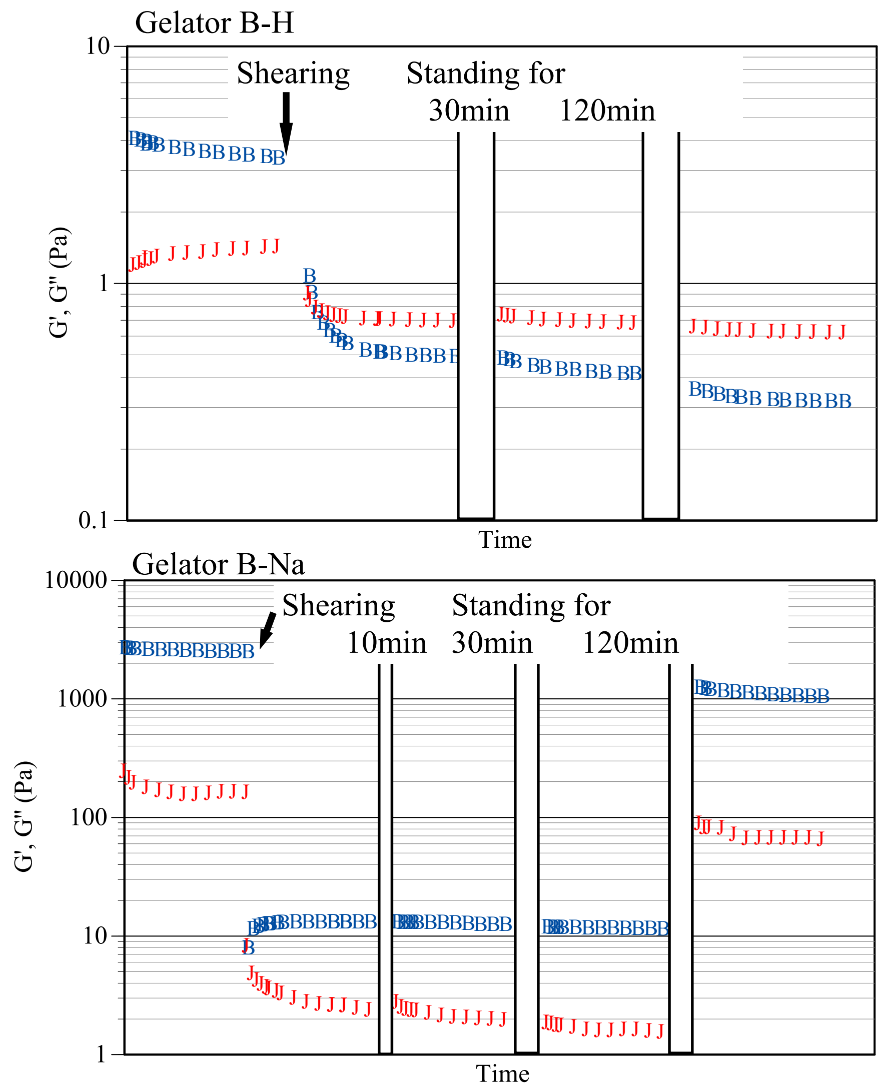

Figure S5. Time dependent rheological analysis of kerosene gels based on gelator B-H (6 mg/mL) and gelator A-Na (8 mg/mL) at a fixed strain (0.05%) and frequency (0.05 Hz).

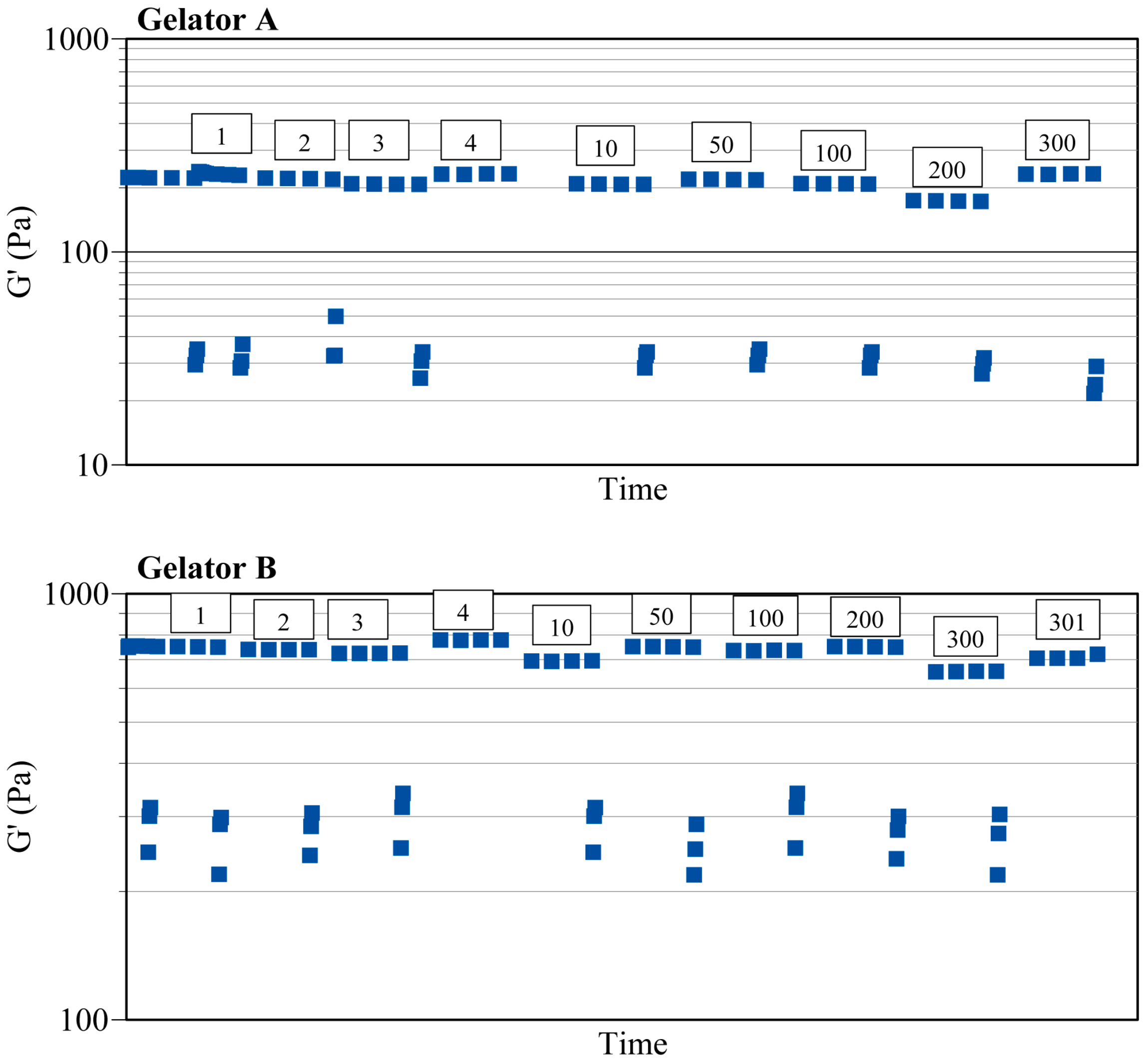

Figure S6. Cyclic time dependent rheological analysis of kerosene gels based on gelator A (10 mg/mL) and gelator B (15 mg/mL) at a fixed strain (0.05%) and frequency (0.05 Hz). The standing time is 30 min. The numbers in Figure indicate the cycle numbers of measurement.

For 1–10, 50, 100, 200, 300, and 301 cycles, the measurement was carried out on the rheometer. For the gels in the screw capped test tube, the operation of the shearing by a shaker (10 s) and then standing at 30 min was repeatedly conducted.

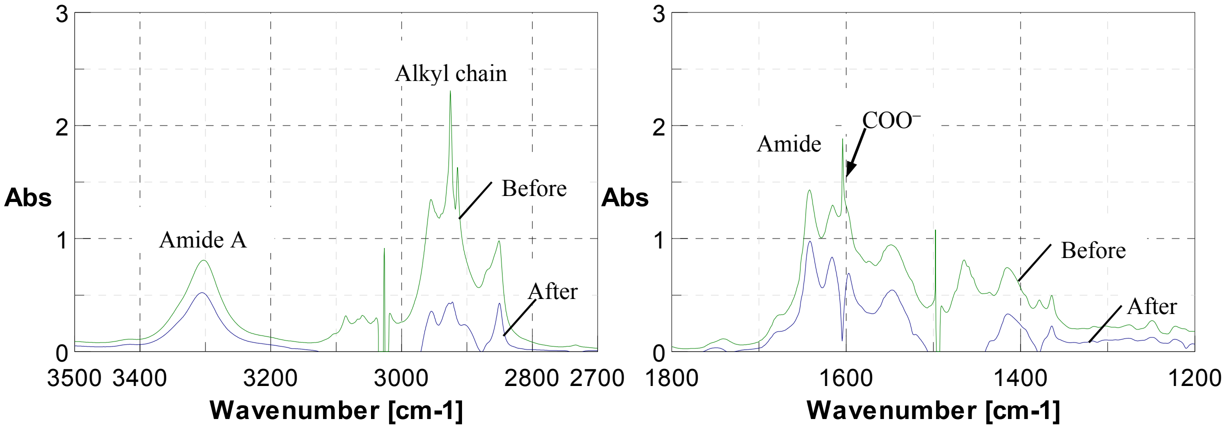

Figure S7. FTIR spectra of toluene gels based on gelator A-Na (20 mg/mL) before and after shearing.

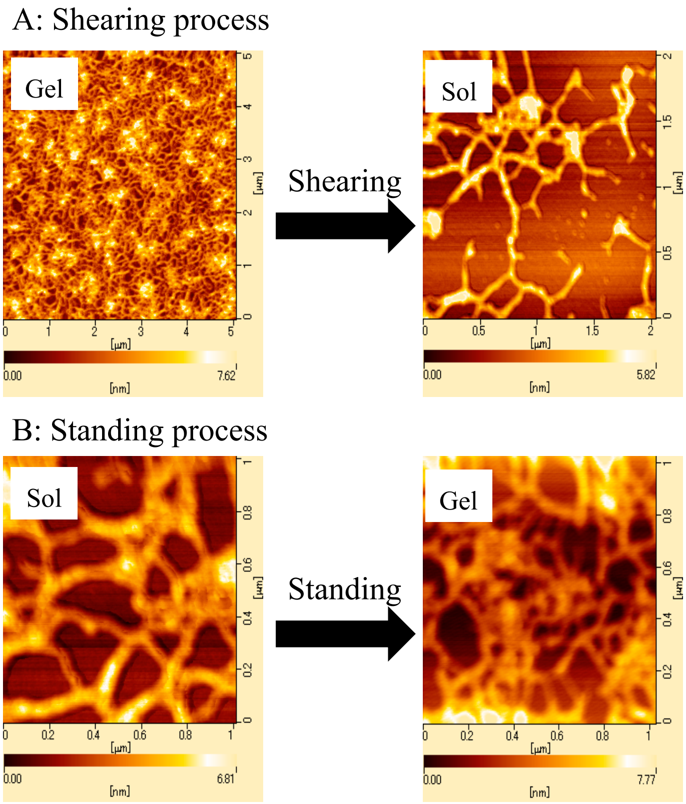

Figure S8. AFM images of kerosene gel based on gelator A (10 mg/mL) in shearing process (A) and standing process (B).

The AFM samples were prepared as follows: the gelator A was dissolved in kerosene (10 mg/mL) in a screw capped test tube by heating, and then the test tube was allowed to stand at 25 °C for 2 h. The gel was broken by shaking (change into the sol), and then the sample was allowed to stand at 25 °C for 30 min. The gel re-formed. The gel was first sample for the AFM, and the AFM sample was prepared on mica by spin coating. The re-formed gel sample in the test tube was broken again by shaking, and the sol was imidiately spin-coated on mica. This is the sol sample. The sol in the test tube was allowed to stand at 25 °C for 30 min, the gel formed again. The second gel sample was prepared from it.

Compared with the images before shearing and after standing, the diameter and amount of the nanofibrous assemblies in the image after shearing became bold and small, respectively. The nanofibers separated from the networks are difficult to fix on mica, leading to the low density of the nanofibers (sample after shearing). Furthermore, the nanofibers may aggregate to the thicker nanofibers during the preparation of AFM sample. In contrast, for the samples prepared from the gels, the network structures can inhibit further aggregation to make the thicker nanofibers and be easy to fix the gel network on mica. The fact is one of the evidences for the degradation of network structures by shearing.

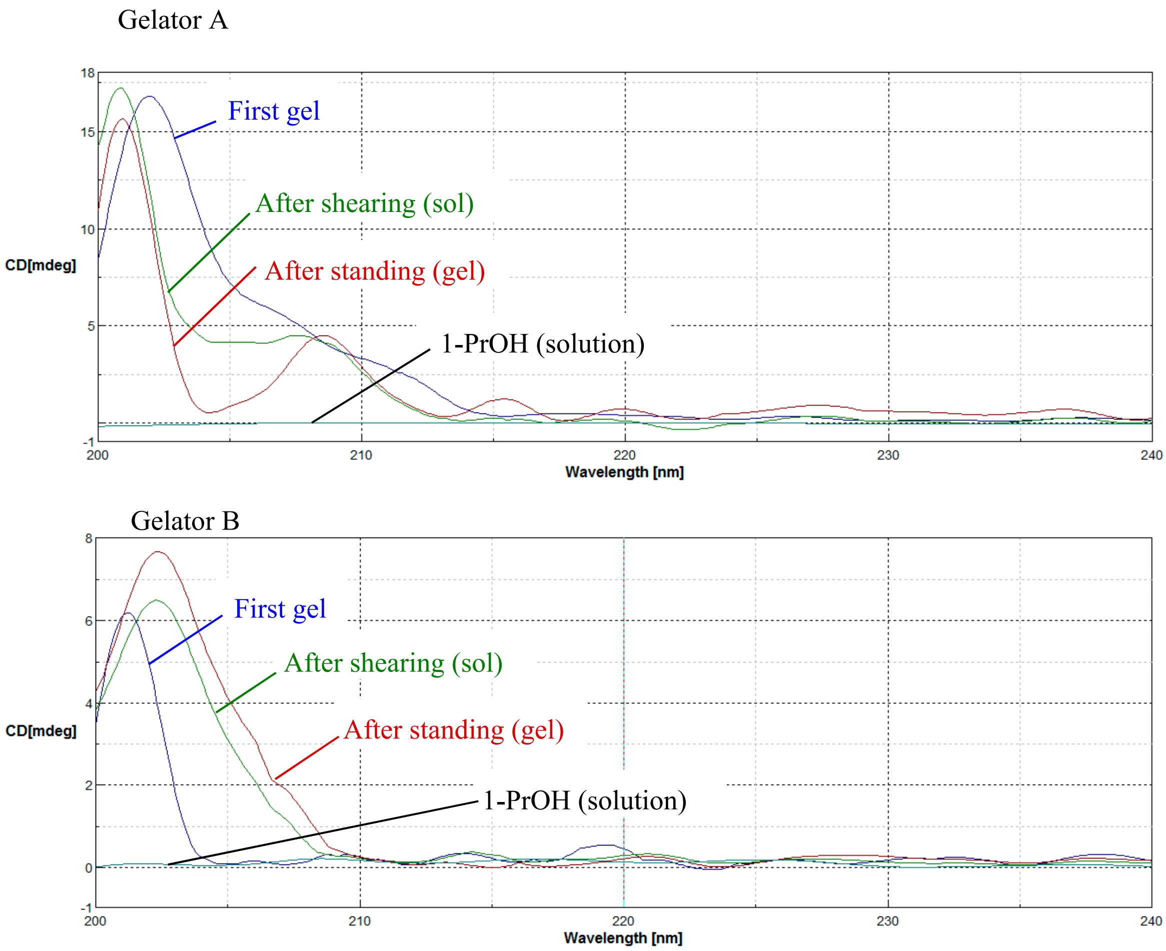

Figure S9. CD spectra of kerosene gels of gelators A (10 mg/mL) and gelators B (15 mg/mL) and their 1-propanol solutions. The first gel means the kerosene gel formed after the heating dissolution of the gelators.

The circular dichroism spectra were measured by using a JASCO Circular Dichroism J–600 spectrometer. In 1-propanol, no CD spectra were obtained because these gelators did not form the gel and have any self-assemblies. For gelator A, the first gel showed the positive CD peak at 202 nm. After shearing, the peak became a little narrow band very slightly shifted to 201 nm. The CD peak of the gel re-formed after standing little changed. For gelator B, the first gel showed the positive CD peak at 201 nm. After shearing, the peak a little broadened and very slightly shifted to 202 nm. The CD peak of the gel re-formed after standing little changed.

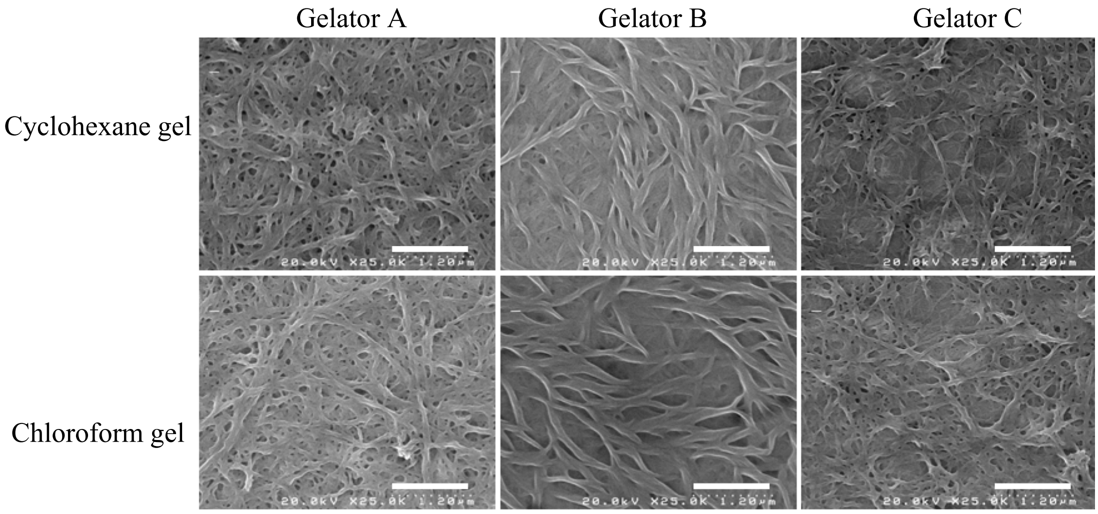

Figure S10. FE-SEM images of dried gels prepared from cyclohexane gels (upper) and chloroform gels (under) of gelators A–C at their MGC. Scale bars are 1.20 µm.

The FE-SEM samples were prepared in vacuum by freeze-dried samples for cyclohexane gel and the room temperature-dried samples for chloroform.

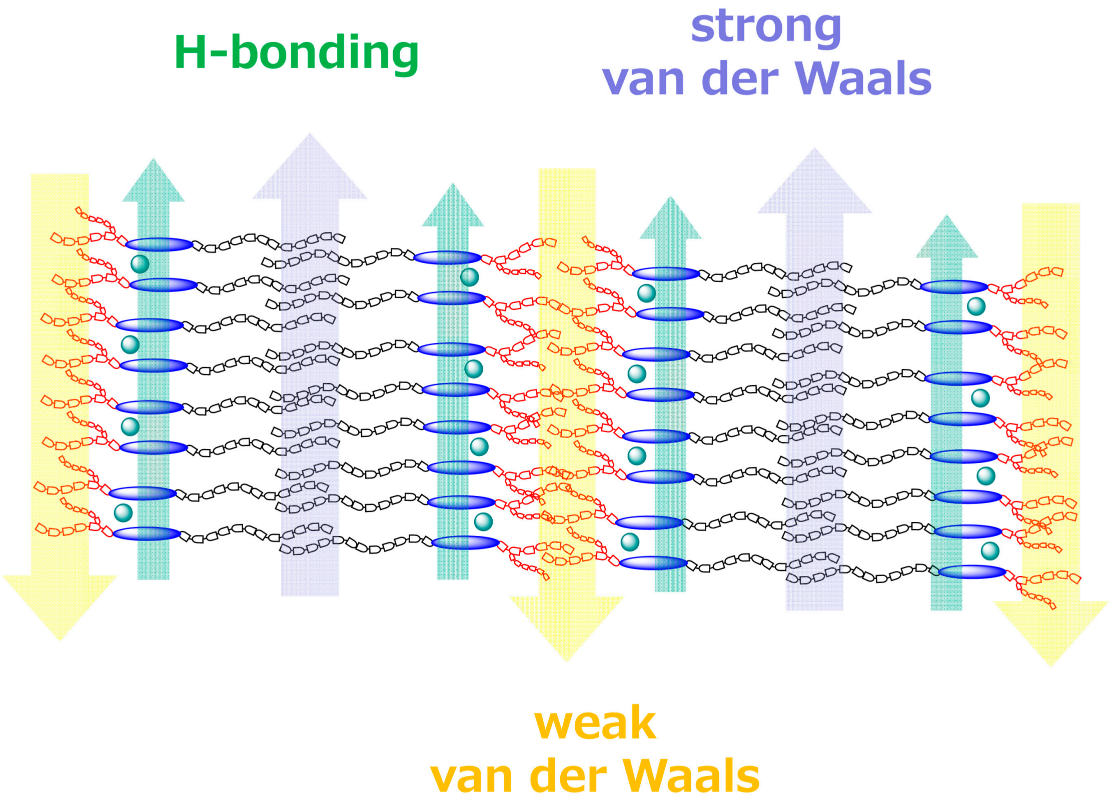

Scheme S1. Tentative illustration of intermolecular interactions in self-assembled nanofibers.
